# Geriatric nutritional risk index as a prognostic marker for patients with upper tract urothelial carcinoma receiving radical nephroureterectomy

**DOI:** 10.1038/s41598-023-31814-2

**Published:** 2023-03-20

**Authors:** Li-Wen Chang, Sheng-Chun Hung, Chuan-Shu Chen, Jian-Ri Li, Kun-Yuan Chiu, Shian-Shiang Wang, Cheng-Kuang Yang, Kevin Lu, Cheng-Che Chen, Shu-Chi Wang, Chia-Yen Lin, Chen-Li Cheng, Yen-Chuan Ou, Shun-Fa Yang

**Affiliations:** 1 Institute of Medicine, Chung Shan Medical University, No. 110, Sec.1, Jianguo N. Rd., Taichung, 40201 Taiwan, ROC; 2 Division of Urology, Department of Surgery, Taichung Veterans General Hospital, No. 1650, Sec. 4, Taiwan Boulevard, Taichung, Taiwan, ROC; 3 Department of Post-Baccalaureate Medicine, College of Medicine, National Chung Hsing University, Taichung, Taiwan, ROC; 4 Department of Medicine and Nursing, Hungkuang University, Taichung, Taiwan, ROC; 5 Department of Applied Chemistry, National Chi Nan University, Nantou, Taiwan; 6 School of Medicine, National Yang Ming University, Taipei, Taiwan; 7 Department of Medical Research, Taichung Veterans General Hospital, Taichung, Taiwan, ROC; 8 Department of Urology, Tungs’ Taichung MetroHarbor Hospital, Taichung, Taiwan, ROC; 9 Department of Medical Research, Chung Shan Medical University Hospital, Taichung, Taiwan

**Keywords:** Cancer, Urological cancer

## Abstract

To investigate the prognostic value of the geriatric nutritional risk index (GNRI) in patients with upper tract urothelial cell carcinoma (UTUC) receiving radical nephroureterectomy (RNU). Between January 2001 and December 2015, we enrolled 488 patients with UTUC underwent RNU in Taichung Veterans General Hospital. GNRI before radical surgery was calculated based on serum albumin level and body mass index. The malnutritional status was defined as GNRI < 92.0. Using Kaplan–Meier analyses and Cox proportional hazards models to analyze the risk factors on disease-free survival (DFS), cancer-specific survival (CSS) and overall survival (OS). 386 patients were categorized as normal nutritional status (GNRI ≥ 92) and 102 patients as malnutritional status (GNRI < 92). We used the receiver operating characteristic (ROC) curve for determined the association between GNRI and OS, with area under the curve (AUC) being 0.69. The 5-year survival rate of DFS, CSS and OS were 48.6%, 80.5% and 80.5% in the normal nutritional group and 28.0%, 53.2% and 40% in the malnutritional group. Using the multivariate analysis, malnutritional status was found as an independent risk factor for OS (hazard ratio [HR] = 3.94, 95% confidence interval [CI] 2.70–5.74), together with age (HR = 1.04, 95% CI 1.02–1.06), surgical margin positive (HR = 1.78, 95% CI 1.13–2.82), pathological T3 (HR = 2.54, 95% CI 1.53–4.21), pathological T4 (HR = 6.75, 95% CI 3.17–14.37) and lymphovascular invasion (HR = 1.81, 95% CI 1.16–2.81). We also found GNRI index as independent risk factor in DFS (HR = 1.90, 95% CI 1.42–2.54) and CSS (HR = 5.42, 95% CI 3.24–9.06). Preoperative malnutritional status with low GNRI is an independent marker in predicting DFS, CSS and OS in UTUC patients underwent RNU.

## Introduction

Urothelial carcinoma (UC) is a urothelial originated malignant disease, involving mostly the low urinary tract (bladder and urethral) while upper tract urothelial carcinoma (UTUC) is relative uncommon, accounting for only 5–10% of UCs^1^. Compared with western countries, the incidence of UTUC in Taiwan is much higher due to arsenic water contamination, herb consumption and prevalence hemodialysis, constituting 40.2% of all UCs^2,3^.

Radical nephroureterectomy (RNU) with bladder cuff excision is the standard treatment for clinical localized UTUCs^4^. Despite staging and surgical refinements, oncology outcome after RNU remain unchanged over the past decades^5^. Tumor stage and grade are the main prognostic factors. Analyses of the SEER database on 5-year cancer specific survival (CSS) showed 86% for T1N0, 77% for T2N0, 63% for T3N0 and 39% for locally advanced^6^. Other tumor-related factors that affect oncology outcomes include variant histology, lymph node involvement, lymphovascular invasion, surgical margins, extensive tumor necrosis and hydronephrosis^7^.

Patients’ factors such as comorbidity, American Society of Anesthesiologists (ASA), performance status (PS) and Charlson Comorbidity Index are also associated with survival outcome on top of the disease stage^8,9^.

Malnutrition is a common problem in cancer patients that may progress to cachexia, leading to poor response to therapy, relative poor prognosis and lower quality of life^10^. The geriatric nutritional risk index (GNRI), which consists of serum albumin level and the ratio of actual and ideal body weights, is a simple and accurate screening method initially designed to predict outcomes in hospitalized elderly patients^11^. Low GNRI is associated with poor prognosis in many human malignancies regarding less treatment response and shorter survival time^12^. The index could also be used in predicting perioperative and oncological outcomes for patients with esophageal cancer, gastric cancer, colorectal cancer, bladder cancer and kidney cancer who have received definitive radical surgery^13–17^. In metastatic urothelial carcinoma, GNRI index is known to be a useful predictive biomarker for chemotherapy and immune checkpoint inhibitors and poor nutrition status associated with less treatment response and survival^18–20^.

No study has yet been published regarding the association between the GNRI and localized UTUC. Here, we aim to investigate the impact of GNRI on survival outcomes of UTUCs receiving RNU.

## Patients and methods

### Patient selection

This study was retrospective chart reviewed analysis and it was approved by the Institutional Review Broad of Taichung Veterans General Hospital (IRB No. CE13240A-3) and informed consents were obtained from all participants. All methods were performed in accordance with the relevant guidelines and regulations. From 2001 to 2015, 728 patients with pathological confirmed UTUC underwent RNU with bladder cuff excision at Taichung Veterans General Hospital. Initially, 520 patients with primary UTUC and available medical record were included in the study. 13 patients were excluded due to loss of follow-up within the first year after operation and 2 patients were excluded due to died related to surgery. 15 patients were excluded due to concurrent radical cystectomy and 2 patients were excluded due to no albumin report. Finally, 488 patients were enrolled in the analysis.

RNU approached included traditional open nephroureterectomy through thoracoabdominal incision (n = 67), laparoscopic transperitoneal nephroureterectomy (n = 403) and retroperitoneoscopy nephroureterectomy (n = 18). We performed hilar lymph node dissection only in patients clinically suspicious lymph node metastasis before 2007. Since 2008, hilar lymph node dissection with or without regional lymph node dissection was routinely performed during RNU. The templates of regional lymph node dissection included para-aortic and peri-caval lymph node for renal pelvis and proximal ureter tumor and pelvic lymph node for distal ureter tumor. Adjuvant chemotherapies with cisplatin-based regimens were performed for those with advanced tumor feature (T3/4 or lymph node positive) but not routinely practiced according to clinicians preference^21^.

Tumor staging followed the American Joint Committee on Cancer and the International Union for Cancer Control updated tumor-node-metastasis (TNM) cancer staging system^22^. Tumor grade was determined in accordance with the 2004 World Health Organization/International Society of Urologic Pathology consensus classification^23,24^.

### Surveillance protocol

All patients were under periodic monitoring protocol: every 3 months during the first two years after operation, every 3 or 6 months during the third year in the case of no evidence of recurrence or progression. The follow-up protocol included laboratory studies, urine cytology, computed tomography (or magnetic resonance imaging) and cystoscope evaluation.

### The geriatric nutritional risk index

The nutritional status with GNRI values was calculated as follows: GNRI = 1.489 × serum albumin level (g/L) + 41.7 × (actual body weight [kg]/ideal body weight [kg])^25^. The ideal body weight was identified as [height (m)]^2^ × 22 (kg/m^2^). The value of the actual body weight/ideal body weight was set to 1 when the actual body weight exceeded the ideal body weight. Malnutritional status was defined as a GNRI < 92.0, according to previous literature^15,20^. Patients were divided into either the normal nutrition group (GNRI ≥ 92.0) and malnutrition group (GNRI < 92.0).

### Patient characteristics

Patient characteristics included the following: gender, age at operation, Eastern Cooperative Oncology Group (ECOG) performance status, albumin, hypertension, diabetes mellitus, coronary artery disease, Body Mass Index (BMI, kg/m^2^), smoking status, renal function, surgical modality, tumor location, surgical margin status, pathological TNM stage, tumor grade, concomitant carcinoma in situ (CIS), lymphovascular invasion and adjuvant chemotherapy.

### Outcome assessment and statistical analysis

End point assessment included: Disease Free Survival (DFS), Cancer Specific Survival (CSS) and Overall Survival (OS), as counted from the date of the RNU. Receiver operating characteristic (ROC) curve was used to determine the cut-off value for overall survival using the Youden index. Mann–Whitney U test was used for continuous variables, and Pearson’s chi-squared test was used for categorical variables. The Kaplain − Meier survival curve and log-rank test was used to determine survival outcomes. For the association between the variables, we used univariate and multivariate Cox hazard regression models to analyze the hazard ratio (HR) and 95% Confidence Interval (CI). Analyses were conducted using the Statistical Package for Social Sciences (SPSS), version 22.0.

### Ethics statement

The studies involving human participants were reviewed and approved by certification at Taichung Veteran General Hospital, Taiwan, with Certification of approval with IRB: CE13240A-3. The patients/participants provided their written informed consent to participate in this study.

## Results

### Patient and characteristics

A total of 488 patients were enrolled in the study.102 patients were in the malnutrition group (GNRI < 92) and 386 patients were in the normal nutrition group (GNRI ≥ 92) (Table 1). The median age was 70.0 years (range 63.8–76.0) in the malnutrition group, and 67.0 years (58.0–76.0) in the normal nutrition group (*p* = 0.023). The median GNRI was 86.8 (range 83.4–89.3) in the malnutrition group, and 101.3 (range 96.9–104.3) in the normal nutrition group (*p* < 0.001). There was no statistical difference between the two groups in terms of comorbidity, smoking status, preoperative renal function, history of uremia, surgical modality and tumor location. The median follow-up period was 23.2 months (range 11.3–36.1) in the malnutrition group and 41.2 months (range 27.0–65.0) in the normal nutrition group (*p* < 0.001).

**Table 1:** Demographic (N = 488).

	GNRI < 92 (n = 102)	GNRI ≥ 92 (n = 386)	*P*-value
Gender			0.846
Male	44 (43.1%)	160 (41.5%)	
Female	58 (56.9%)	226 (58.5%)	
Age	70.0 (63.8–76.0)	67.0 (58.0–76.0)	0.023*
BMI (kg/m^2^)	22.9 (19.9–24.7)	24.2 (21.8–26.2)	< 0.001**
Albumin (g/dL)	3.1 (2.9–3.3)	4.0 (3.8–4.3)	< 0.001**
GNRI	86.8 (83.4–89.3)	101.3 (96.9–104.3)	< 0.001**
Performance status ECOG			0.002**
0	14 (13.7%)	49 (12.7%)	
1	58 (56.9%)	279 (72.3%)	
2–4	30 (29.4%)	58 (15.0%)	
**Comorbidity**			
HTN	65 (63.7%)	233 (60.4%)	0.613
DM	27 (26.5%)	78 (20.2%)	0.217
COPD/asthema	5 (4.9%)	12 (3.1%)	0.369
CAD	6 (5.9%)	13 (3.4%)	0.252
Creatinine > 1.5 mg/dL	30 (29.4%)	99 (25.6%)	0.522
HBV or HCV carrier	14 (13.7%)	42 (10.9%)	0.531
Previous UCUB	21 (20.6%)	66 (17.1%)	0.501
Hydronephrosis	11 (10.8%)	39 (10.1%)	0.986
Smoking status			0.695
Never	77 (75.5%)	284 (73.6%)	
Current/former	25 (24.5%)	102 (26.4%)	
Preoperative renal function			0.157
eGFR ≥ 30 ml/min/1.73m^2^	71 (69.6%)	295 (76.4%)	
eGFR < 30 ml/min/1.73m^2^	31 (30.4%)	91 (23.6%)	
History of uremia			0.887
Negative	86 (84.3%)	330 (85.5%)	
Positive	16 (15.7%)	56 (14.5%)	
Surgical modality			0.187
Open	19 (18.6%)	48 (12.4%)	
Transperitoneal laparoscopy	78 (76.5%)	325 (84.2%)	
Retroperitoneoscopy	5 (4.9%)	13 (3.4%)	
**Tumor location**			
Calyx	24 (23.5%)	90 (23.3%)	1.000
Renal pelvis	56 (54.9%)	241 (62.4%)	0.203
Promixal ureter	40 (39.2%)	120 (31.1%)	0.151
Middle ureter	29 (28.4%)	80 (20.7%)	0.126
Distal ureter	22 (21.6%)	97 (25.1%)	0.538
Surgical margin			0.026*
Negative	85 (83.3%)	353 (91.5%)	
Positive	17 (16.7%)	33 (8.5%)	
Pathological T			0.067
T1	46 (45.1%)	204 (52.8%)	
T2	13 (12.7%)	46 (11.9%)	
T3	30 (29.4%)	115 (29.8%)	
T4	13 (12.7%)	21 (5.4%)	
Pathological N			< 0.001**
N0	83 (81.4%)	362 (93.8%)	
N1	5 (4.9%)	11 (2.8%)	
N2–3	14 (13.7%)	13 (3.4%)	
Tumor grade			0.090
Low	5 (4.9%)	40 (10.4%)	
High	97 (95.1%)	346 (89.6%)	
Concomitant CIS			0.082
Negative	79 (77.5%)	329 (85.2%)	
Positive	23 (22.5%)	57 (14.8%)	
Lymphovascular invasion			0.001**
Negative	67 (66.3%)	314 (81.3%)	
Positive	35 (34.3%)	72 (18.7%)	
Adjuvant Chemotherapy	25 (24.5%)	92 (23.8%)	0.991
F/u time (month)	23.2 (11.3–36.1)	41.2 (27.0–65.0)	< 0.001**

*GNRI* geriatric nutritional risk index, *BMI* body mass index, *ECOG* Eastern Cooperative Oncology Group, *HTN* hypertension, *DM* diabetes mellitus, *COPD* chronic obstructive pulmonary disease, *CAD* coronary artery disease, *HBV* Hepatitis B virus, *HCV* Hepatitis C virus, *UCUB* urothelial carcinoma in urinary bladder, *eGFR* estimated Glomerular filtration rate, *CIS* carcinoma in situ.

Chi-square test. Mann–Whitney Test, Median (IQR). *P < 0.05, **P < 0.01.

We hypothesized poor nutrition with low GNRI not only result from the patient himself but also the sequela of the more advanced malignant disease. In Table 1, we found that more advanced tumor in malnutrition group than in normal nutrition group such as pathological T stage (*p* = 0.067), pathological N stage (*p* < 0.001), positive surgical margin (16.7% vs. 8.5%, *p* = 0.026) and lymphovascular invasion (34.3% vs. 18.7%, *p* = 0.001). Additionally in supplementary Fig. 1 to 4, we found that high pathological T stage, high pathological N stage, lymphovascular invasion and surgical margin positive were associated with lower GNRI index score.

### GNRI cut-off value

**Figure 1:**
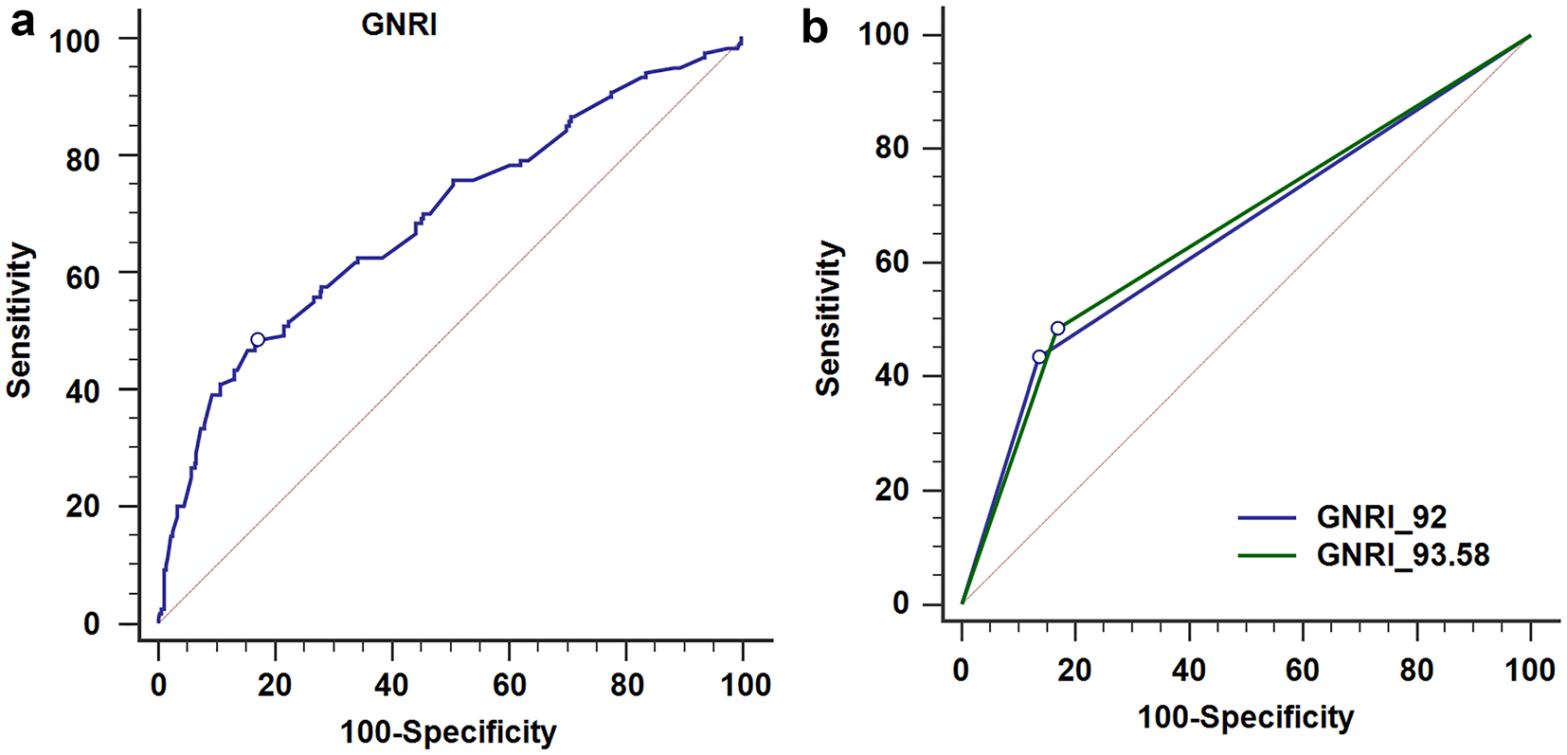
ROC for GNRI as a predictive factor for overall survival. The cut-off value was 93.58 with an AUC of 0.69 (sensitivity: 0.48, specificity: 0.83).

The ROC curve was plotted for GNRI as a predictive factor for OS and revealed the area under curve (AUC) was 0.69 with a cut-off value 93.58 months (Fig. 1a). When using the cut-off value as 93.58 months, the sensitivity was 48.33% and the specificity was 83.15% (AUC 0.657). When using the cut-off value as 92, the sensitivity was 43.33% and the specificity was 86.41% (AUC 0.649), respectively. We used Delong test to examine the two cut-off and showed no significant difference in predicting overall survival (*p* = 0.430) (Fig. 1b; Table 2). Thus, we think GNRI cut-off value 92 and 93.58 were both efficacy for patients with UTUC receiving RNU. Additionally, we used the cut-off value of 93.58 to categorize patient characteristics and their demographic as shown in Supplementary Table 1 and to exam the predict value in Fig. 3.

**Table 2:** Sensitivity and specificity of different GNRI cut-off value.

Variables	AUC	(95% CI)	*DeLong test p*	Sensitivity (%)	Specificity (%)
GNRI 92	0.649	(0.61–0.69)	0.430	43.33	86.41
GNRI 93.58	0.657	(0.61–0.70)		48.33	83.15

Outcome: death.

### Overall survival

**Figure 2:**
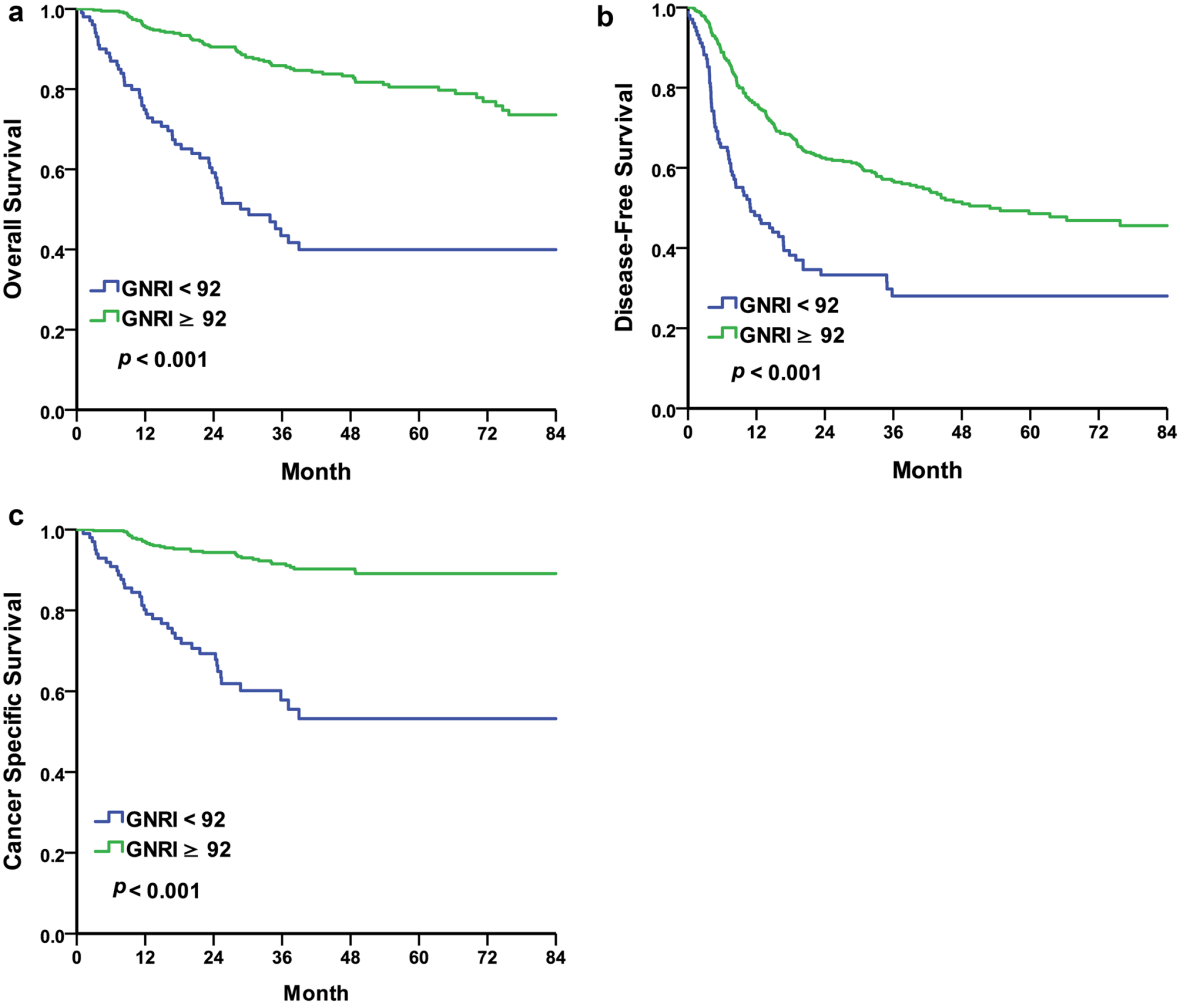
Kaplan–Meier curve for UTUC patients comparing normal nutrition group and malnutrition group according to GNRI = 92. (**a**) Overall survival, median 30.16 months in malnutrition group and half of patients were survive in normal nutrition group, *p* < 0.001. (**b**) Disease free survival, median 10.97 months in malnutrition group and 52.93 months in normal nutrition group, *p* < 0.001. (**c**) Cancer specific survival, median time were not calculated due to half of patients were survive in both group, *p* < 0.001.

**Figure 3:**
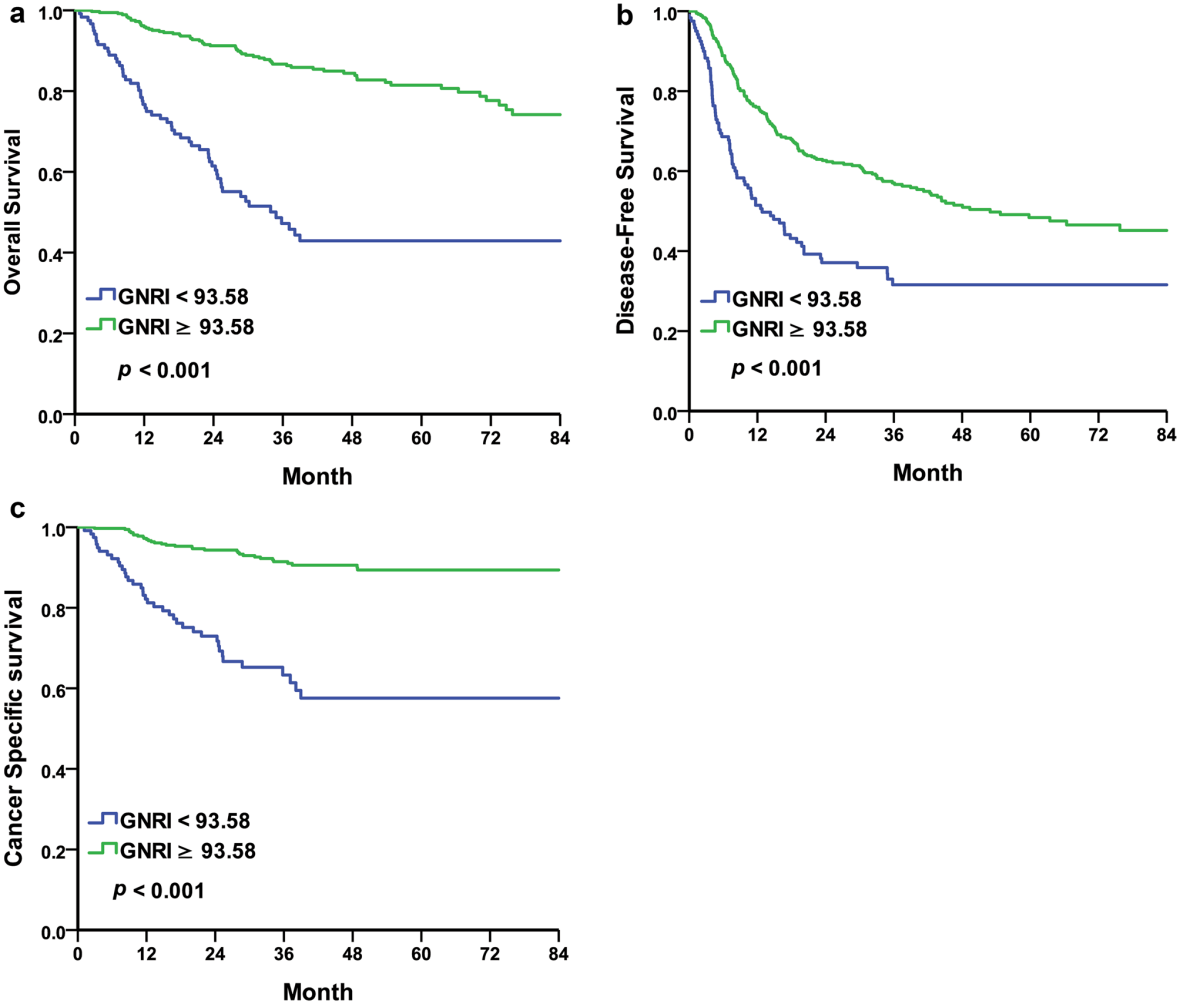
Kaplan–Meier curve for UTUC patients according to cut-off value of GNRI by 93.58. (**a**) Overall survival, median 34.83 months in GNRI < 93.58 and half of patients were survive in GNRI ≥ 93.58, *p* < 0.001. (**b**) Disease free survival, median 12.81 months in GNRI < 93.58 and 52.93 months in GNRI ≥ 93.58, *p* < 0.001. (**c**) Cancer specific survival, median time were not calculated due to half of patients were survive in both group, *p* < 0.001.

We compared OS between the malnutrition and normal nutrition groups. Using GNRI 92 as cut-off value, the median OS was 30.16 months in the malnutrition group and the median OS could not be calculated due to half of patients were survive in the normal nutrition group (*p* < 0.001) (Fig. 2a). Using GNRI 93.58 as the cut-off value, the median OS was 34.83 months in patients with GNRI < 93.58 and half of patients were survived in GNRI ≥ 93.58 (*p* < 0.001) (Fig. 3a). Univariate and multivariate analyses with COX regression revealed GNRI < 92 being an independent risk factor for OS (HR = 3.94, 95% CI 2.70–5.74, *p* < 0.001) (Table 3). We also found other independent risk factors for OS including the following: age (HR = 1.04, 95% CI 1.02–1.06, *p* < 0.001), surgical margin positivity (HR = 1.78, 95% CI 1.13–2.82, *p* = 0.013), pathological T3 (HR = 2.54, 95% CI 1.53–4.21, *p* < 0.001), pathological T4 (HR = 6.75, 95% CI 3.17–14.37, *p* < 0.001) and lymphovascular invasion (HR = 1.81, 95% CI 1.16–2.81, *p* = 0.008).

**Table 3:** Cox regression—overall survival.

	Univariate			Multivariable		
	Hazard ratio	95% CI	*p-*value	Hazard ratio	95% CI	*p-*value
**Gender**						
Female	Reference	Reference		Reference	Reference	
Male	1.44	(1.01–2.06)	0.046*	0.75	(0.52–1.09)	0.135
Age	1.03	(1.02–1.05)	< 0.001**	1.04	(1.02–1.06)	< 0.001**
**GNRI**						
≥ 92	Reference	Reference		Reference	Reference	
< 92	4.47	(3.10–6.43)	< 0.001**	3.94	(2.70–5.74)	< 0.001**
**Performance status ECOG**						
0	Reference	Reference				
1	0.73	(0.44–1.20)	0.209			
2–4	1.43	(0.82–2.51)	0.209			
**Comorbidity**						
HTN	1.09	(0.75–1.58)	0.647			
DM	1.57	(1.05–2.33)	0.027*	0.92	(0.60–1.41)	0.699
COPD/asthema	1.75	(0.72–4.30)	0.220			
CAD	1.89	(0.92–3.87)	0.082			
Creatinine > 1.5 mg/dL	1.38	(0.94–2.05)	0.104			
**Smoking status**						
Never	Reference	Reference				
Current/former	1.39	(0.95–2.03)	0.094			
**Preoperative renal function**						
eGFR ≥ 30 ml/min/1.73m^2^	Reference	Reference				
eGFR < 30 ml/min/1.73m^2^	1.42	(0.96–2.10)	0.077			
**History of uremia**						
Negative	Reference	Reference				
Positive	1.19	(0.73–1.92)	0.487			
**Surgical margin**						
Negative	Reference	Reference		Reference	Reference	
Positive	4.62	(3.05–7.02)	< 0.001**	1.78	(1.13–2.82)	0.013*
**Pathological T**						
T1	Reference	Reference		Reference	Reference	
T2	2.33	(1.20–4.52)	0.013*	1.51	(0.77–2.99)	0.232
T3	4.19	(2.64–6.64)	< 0.001**	2.54	(1.53–4.21)	< 0.001**
T4	18.21	(10.43–31.79)	< 0.001**	6.75	(3.17–14.37)	< 0.001**
**Pathological N**						
N0	Reference	Reference		Reference	Reference	
N1	3.15	(1.53–6.50)	0.002**	1.23	(0.57–2.63)	0.597
N2–3	6.27	(3.84–10.22)	< 0.001**	1.08	(0.56–2.09)	0.811
**Tumor grade**						
Low	Reference	Reference		Reference	Reference	
High	15.65	(2.18–112.03)	0.006**	7.22	(0.99–52.96)	0.052
**Lymphovascular invasion**						
Negative	Reference	Reference		Reference	Reference	
Positive	4.39	(3.06–6.31)	< 0.001**	1.81	(1.16–2.81)	0.008**

*GNRI* geriatric nutritional risk index, *ECOG* Eastern Cooperative Oncology Group, *HTN* hypertension, *DM* diabetes mellitus, *COPD* chronic obstructive pulmonary disease, *CAD* coronary artery disease, *eGFR* estimated Glomerular filtration rate.

Cox proportional hazard regression. *p < 0.05, **p < 0.01.

### Disease free survival

Using GNRI 92 as the cut-off value, the median DFS was 10.97 months in the malnutrition group, and 52.93 months in the normal nutrition group, (*p* < 0.001) (Fig. 2b). Using GNRI 93.58 as the cut-off value, the median DFS was 12.81 months in patients with GNRI < 93.58 and was 52.93 months in patients with GNRI ≥ 93.58 (*p* < 0.001) (Fig. 3b). Univariate and multivariate analyses with COX regression revealed GNRI < 92 as an independent risk factor of DFS (HR = 1.90, 95% CI 1.42–2.54, *p* < 0.001) (Table 4). We also found other independent risk factors for DFS including the following: surgical margin positivity (HR = 1.68, 95% CI 1.13–2.49, *p* = 0.010), pathological T4 (HR = 1.89, 95% CI 1.06–3.37, *p* = 0.031) and lymphovascular invasion (HR = 1.67, 95% CI 1.19–2.35, *p* = 0.003).

**Table 4:** Cox regression—disease free survival.

	Univariate			Multivariable		
	Hazard ratio	95% CI	*p-*value	Hazard ratio	95% CI	*p-*value
**Gender**						
Female	Reference	Reference		Reference	Reference	
Male	1.38	(1.08–1.77)	0.011*	1.22	(0.94–1.58)	0.131
Age	1.01	(1.00–1.02)	0.090			
**GNRI**						
≥ 92	Reference	Reference		Reference	Reference	
< 92	2.19	(1.65–2.90)	< 0.001**	1.90	(1.42–2.54)	< 0.001**
**Performance status ECOG**						
0	Reference	Reference				
1	1.15	(0.78–1.68)	0.484			
2–4	1.35	(0.86–2.11)	0.193			
**Comorbidity**						
HTN	1.02	(0.79–1.31)	0.898			
DM	1.36	(1.02–1.82)	0.036*	1.19	(0.88–1.61)	0.271
COPD/asthema	2.12	(1.21–3.71)	0.009**	2.06	(0.98–3.70)	0.113
CAD	1.28	(0.73–2.23)	0.394			
Creatinine > 1.5 mg/dL	1.28	(0.97–1.69)	0.078			
**Smoking status**						
Never	Reference	Reference				
Current/former	1.13	(0.86–1.50)	0.372			
**Preoperative renal function**						
eGFR ≥ 30 ml/min/1.73m^2^	Reference	Reference				
eGFR < 30 ml/min/1.73m^2^	1.01	(0.76–1.35)	0.942			
**History of uremia**						
Negative	Reference	Reference				
Positive	1.08	(0.77–1.53)	0.644			
**Surgical margin**						
Negative	Reference	Reference		Reference	Reference	
Positive	3.11	(2.22–4.36)	< 0.001**	1.68	(1.13–2.49)	0.010*
**Pathological T**						
T1	Reference	Reference		Reference	Reference	
T2	1.35	(0.89–2.02)	0.154	1.15	(0.75–1.75)	0.520
T3	1.67	(1.25–2.22)	< 0.001**	1.14	(0.82–1.60)	0.436
T4	4.46	(2.94–6.76)	< 0.001**	1.89	(1.06–3.37)	0.031*
**Pathological N**						
N0	Reference	Reference		Reference	Reference	
N1	1.64	(0.89–3.01)	0.110	0.99	(0.53–1.86)	0.984
N2–3	2.90	(1.90–4.44)	< 0.001**	1.14	(0.68–1.89)	0.626
**Tumor grade**						
Low	Reference	Reference		Reference	Reference	
High	2.37	(1.35–4.14)	0.003**	1.61	(0.90–2.89)	0.110
**Lymphovascular invasion**						
Negative	Reference	Reference		Reference	Reference	
Positive	2.53	(1.94–3.31)	< 0.001**	1.67	(1.19–2.35)	0.003**

*GNRI* geriatric nutritional risk index, *ECOG* Eastern Cooperative Oncology Group, *HTN* hypertension, *DM* diabetes mellitus, *COPD* chronic obstructive pulmonary disease, *CAD* coronary artery disease, *eGFR* estimated Glomerular filtration rate.

Cox proportional hazard regression. *p < 0.05, **p < 0.01.

### Cancer specific survival

Using GNRI 92 as the cut-off value, the median CSS could not be calculated due to half of patients were survive in both group and showed significant difference (*p* < 0.001) (Fig. 2c). Similar result was found when using GNRI 93.58 as the cut-off value (*p* < 0.001) (Fig. 3c). Univariate and multivariate analyses with COX regression revealed GNRI < 92 as an independent risk factor of CSS (HR = 5.42, 95% CI 3.24–9.06, *p* < 0.001) (Table 5). We also found other independent risk factors for CSS including the following: male gender (HR = 1.93, 95% CI 1.15–3.23, *p* = 0.012), age (HR = 1.04, 95% CI 1.01–1.07, *p* = 0.005), surgical margin positivity (HR = 2.16, 95% CI 1.26–3.71, *p* = 0.005), pathological T3 (HR = 6.30, 95% CI 2.57–15.43, *p* < 0.001) and pathological T4 (HR = 24.79, 95% CI 8.21–74.87, *p* < 0.001).

**Table 5:** Cox regression—cancer specific survival.

	Univariate			Multivariable		
	Hazard ratio	95% CI	*p-*value	Hazard ratio	95% CI	*p-*value
**Gender**						
Female	Reference	Reference		Reference	Reference	
Male	1.90	(1.19–3.03)	0.007**	1.93	(1.15–3.23)	0.012*
Age	1.03	(1.00–1.05)	0.022*	1.04	(1.01–1.07)	0.005**
**GNRI**						
≥ 92	Reference	Reference		Reference	Reference	
< 92	6.14	(3.84–9.82)	< 0.001**	5.42	(3.24–9.06)	< 0.001**
**Performance status ECOG**						
0	Reference	Reference				
1	0.48	(0.27–0.88)	0.588			
2–4	1.00	(0.50–1.99)	0.996			
**Comorbidity**						
HTN	1.09	(0.67–1.77)	0.720			
DM	1.05	(0.59–1.86)	0.867			
COPD/asthema	1.66	(0.52–5.28)	0.390			
CAD	1.52	(0.55–4.16)	0.418			
Creatinine > 1.5 mg/dL	1.47	(0.89–2.41)	0.130			
**Smoking status**						
Never	Reference	Reference				
Current/former	1.56	(0.96–2.54)	0.073			
**Preoperative renal function**						
eGFR ≥ 30 ml/min/1.73m^2^	Reference	Reference				
eGFR < 30 ml/min/1.73m^2^	0.79	(0.44–1.42)	0.435			
**History of uremia**						
Negative	Reference	Reference				
Positive	0.55	(0.24–1.27)	0.163			
**Surgical margin**						
Negative	Reference	Reference		Reference	Reference	
Positive	8.31	(5.15–13.41)	< 0.001**	2.16	(1.26–3.71)	0.005**
**Pathological T**						
T1	Reference	Reference		Reference	Reference	
T2	3.26	(1.03–10.29)	0.044*	2.38	(0.72–7.81)	0.154
T3	10.21	(4.54–22.96)	< 0.001**	6.30	(2.57–15.43)	< 0.001**
T4	56.80	(24.07–134.02)	< 0.001**	24.79	(8.21–74.87)	< 0.001**
**Pathological N**						
N0	Reference	Reference		Reference	Reference	
N1	6.23	(2.93–13.24)	< 0.001**	1.50	(0.65–3.47)	0.344
N2–3	11.39	(6.60–19.64)	< 0.001**	1.23	(0.59–2.55)	0.577
**Tumor grade**						
Low	Reference	Reference		Reference	Reference	
High	8.56	(1.19–61.64)	0.033*	1.84	(0.23–14.52)	0.565
**Lymphovascular invasion**						
Negative	Reference	Reference		Reference	Reference	
Positive	7.07	(4.39–11.38)	< 0.001**	1.55	(0.86–2.81)	0.148

*GNRI* geriatric nutritional risk index, *ECOG* Eastern Cooperative Oncology Group, *HTN* hypertension, *DM* diabetes mellitus, *COPD* chronic obstructive pulmonary disease, *CAD* coronary artery disease, *eGFR* estimated Glomerular filtration rate.

Cox proportional hazard regression. *p < 0.05, **p < 0.01.

### Patients age > 70 years old

**Figure 4:**
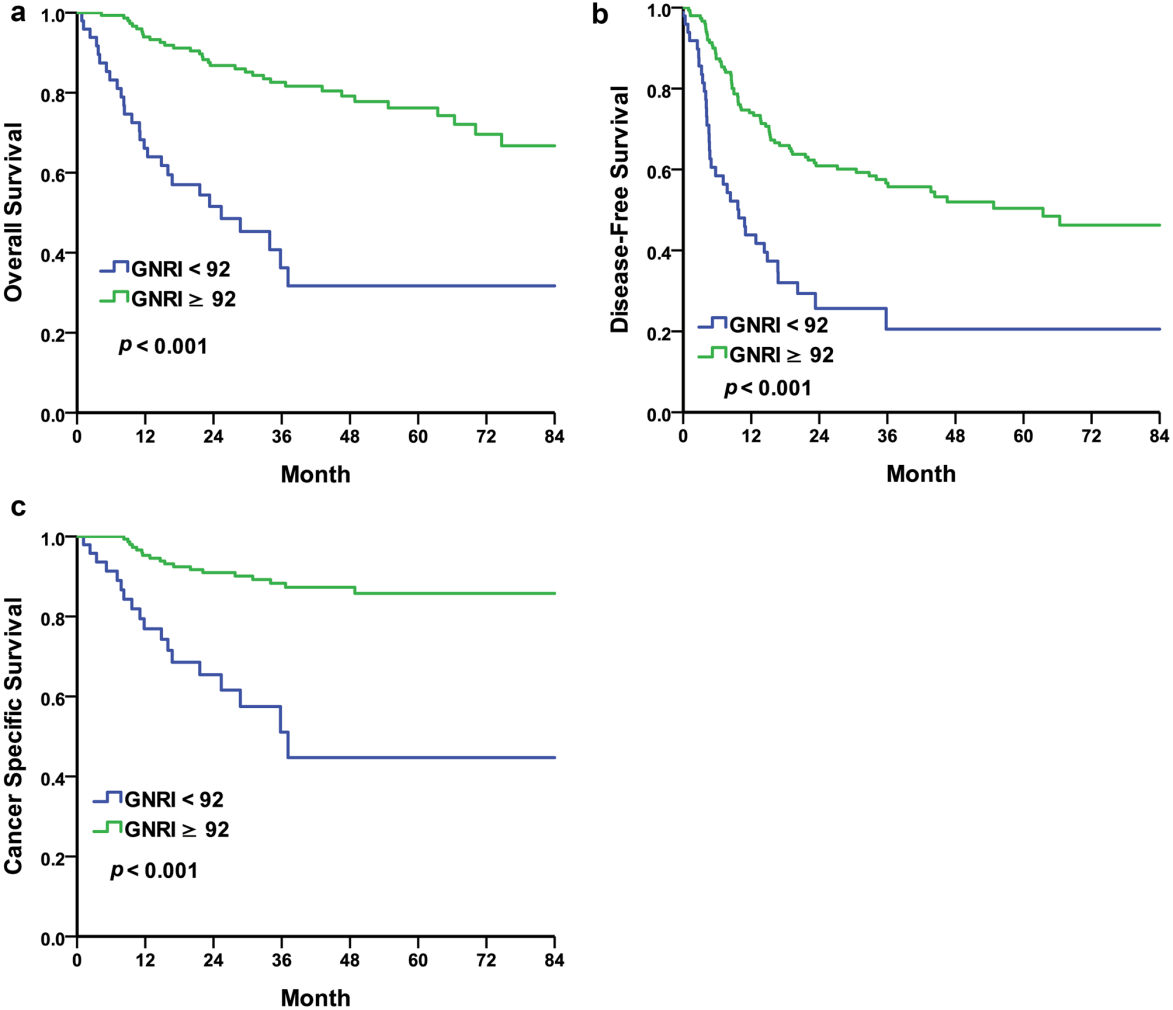
Kaplan–Meier curve for overall survival in UTUC patients elder than 70 years old comparing normal nutrition group and malnutrition group according to GNRI = 92. (**a**) Overall survival, median 25.36 months in GNRI < 92 group and it couldn’t be calculated due to more than half of them were survive in GNRI ≥ 92 group, *p* < 0.001** (**b**) Disease free survival, median 9.79 months in GNRI < 92 group and 63.44 months in GNRI ≥ 92 group, *p* < 0.001** (**c**) Cancer specific survival, median 37.09 months in GNRI < 92 group and it couldn’t be calculated due to more than half of them were survive in GNRI ≥ 92 group, *p* < 0.001**.

Because GNRI index was initially designed for elderly patients, survival analysis was performed in patients elder than 70 years old^11^. In Fig. 4, GNRI index < 92 was associated with shorter OS (p < 0.001, Fig. 4a), DFS (p < 0.001, Fig. 4b) and CSS (p < 0.001, Fig. 4c) in patients age > 70 years old.

### Perioperative complications

Perioperative complications were no significant differences among the two groups. A total 11 complications (13.7%) were found in malnutrition group, and 33 complications (8.5%) in the normal nutrition group (*p* = 0.868) (Supplementary table 2). Among these complications, 3 vascular injuries (2.9%) were in the malnutrition group, and 7 (1.8%) in the normal nutrition group (*p* = 0.506). There were 3 wound infections (2.9%) in the malnutrition group, and 8 (2.1%) in the normal nutrition group (*p* = 0.162) and 8 cases of ileus (7.8%) in the malnutrition group, and 18 (4.7%) in the normal nutrition group (*p* = 1.000).

## Discussion

Our principal finding is that preoperative GNRI, as a nutritional status evaluation tool, is an independent prognostic factor for UTUC patients receiving RNU. Age, surgical margin positive, pathological T stage and lymphovascular invasion are also independently affect the overall survival under a long-term follow-up. Literature suggests that GNRI index < 92 as clinical trigger for nutritional support in institutionalised elderly^26^. We also used GNRI < 92 to verify the prognostic value. Additionally, based on the ROC curve analysis in our study, the cut-off value 93.58 may be more sensitive and specific in predicting survival outcome, although cut-off value 92 may be more convenient to use in clinical practice.

In our analysis, we found that low GNRI index was not only associated with individual physical condition, but also due to advanced tumor behavior. In the supplementary Fig. 1 to 4, we found that advanced pathological features including higher pathological T stage, pathological N stage, surgical margin positive and lymphovascular invasion had significant lower GNRI index value. As the result, this may explain why it was associated with poorer survival outcome.

Malnutrition is a common problem in hospitalized elderly patients, and it is associated with their functional decline and higher mortality rate^27^. Malnutrition may be characterized by loss of muscle or fat mass causing body weight loss, which is common for cancer patients as the result of cachexia and is responsible for 22% of deaths^28^. Pretreatment serum albumin levels provide useful nutritional assessment and prognostic significance for cancer patients. Serum albumin levels may drop due to tumor progression, immune response to tumor and anticancer therapy^29^. The nutritional risk index is first used to evaluate nutritional status and postoperative outcome which is calculated by albumin content, in terms of present body weight and usual body weight^30^. However, this index is not widely used because most elderly patients do not remember their usual body weight, and their weight loss may require correcting multiple contributing factors^31^. GNRI was proposed by Bouillanne et al., and with the usual body weight replaced by ideal body weight. It became a simplified and more convenient predictive tool^11^. Although albumin is a well-known index of nutrition status affecting wound healing and postoperative complication, it will be altered by digestive function and systemic inflammation^32,33^. In contrast, GNRI calculated by albumin, actual body weight and ideal body weight is more objective and easily determined and it was associated with risk of deaths in many human diseases such as diabetes mellitus, cardiovascular disease, end stage renal disease and cancers^34^.

Several studies reported the value of GNRI in predicting oncologic outcome and comorbidity in cancer patients receiving curative surgical treatment. Kubo et al*.,* found that low GNRI are associated with high incidences of preoperative dysphagia, postoperative lung complications and 5-year overall survival in esophagus cancer^13^. Similar to our finding, they found that incidence of nodal metastasis and pathological stage were significantly higher in the GNRI-low group than in the GNRI-high group which contributed to the inferior survival outcomes after esophagectomy. Most of reported literatures focused on the gastrointestinal tract malignancies. Hirahara et al*.* and Sasaki et al*.* also reported that GNRI is an independent prognostic factor for OS in gastric patients underwent laparoscopic gastrectomy and in colorectal cancer patients after curative surgery^14,15^. Significantly higher incidence of postoperative complications was found in low-GNRI group, including surgical site infection, ileus, anastomotic leakage, intra-abdominal abscess, colitis, pneumonia, and urinary infection. The difference of post operative complications was not seen in our population. The possible reason is that no intestinal reconstruction during RNU and anastomotic leakage may cause subsequent complications.

Malnutrition is relative less common in genitourinary tract malignancy, and it may present as a sequela of advanced disease or paraneoplastic syndrome^35^. In a large-scale retrospective study, Kang et al*.* found that low values of GNRI are associated with aggressive pathologic characteristics and poor survival in patients with renal clear cell carcinoma who have nephrectomy^17^. Riveros et al*.* found that in bladder cancer patients receiving radical cystectomy, GNRI independently predicts mortality, blood transfusion, pneumonia, extended length of stay and non-home discharge^16^. Additional to survival and perioperative complications outcome, they also suggest that low GNRI was associated with extended length of hospital stay and nonhome discharge. Moreover, GNRI may being part of Enhanced Recovery after Surgery (ERAS) protocols for nutritional risk screening before radical cystectomy^36^.

Our present study is the first to investigate the relationship between GNRI and survival outcomes in UTUC patients receiving RNU, not only elderly but also young age populations. It could be the sequela of advanced tumor stage causing cancer cachexia or paraneoplastic syndrome and advanced malignant features were associated with low GNRI score. Additionally in multivariate analysis, we confirmed GNRI is the independent risk factor for OS, CSS and DFS.

Treatment protocol for UTUC changed over time. For example, whether performing template lymph node dissection during RNU was inconsistent in our populations. Meta-analysis for retrospective articles suggested templated-based lymph node dissection improve cancer specific survival in high-stage UTUC and reduces the risk of local recurrence^37^. Additionally, population-based cohort studies found that lymph node dissection improved survival outcomes not only in clinical lymph node negative but also pathological lymph node negative UTUC patients^38,39^. Nevertheless, template lymph node dissection was only routinely performed in our populations since 2008 and it may influence the survival outcome. Similarly, the POUT trial suggests that adjuvant gemcitabine-platinum combination chemotherapy significantly improved disease free survival^40^. However, only 23.9% patients in our population received adjuvant chemotherapy and this may influence the conclusion of our analysis.

Below are some limitations of our study. First, retrospective design had selection and information bias that had restricted the power of the prognostic role. Prospective cohort study is needed to overcome the limitations of the potential bias. Second, the surgical method is a possible confounder that impacts oncologic outcomes. The difference of surgical approach and template lymph node dissection may influence the outcome. Third, reports in the literature suggested that the nutrition status is associated with physical performance and the quality of life^41^. However, the research approach using questionnaires was not feasible due to the retrospective nature of our study. Finally, we did not assess the impact of neoadjuvant or adjuvant therapy which may have systemic impacts leading to malnutrition and influence the survival result. In the present era of using immune check-point inhibitors, further large scaled prospective cohort studies are needed to verify the association between GNRI and malignance.

## Conclusions

Preoperative malnutritional status with low GNRI is an independent marker in predicting DFS, CSS and OS in UTUC patients underwent RNU. Age, surgical margin positivity, advanced tumor stage and lymphovascular invasion are also independent prognostic factors.

## Supplementary Information

Supplementary Legends.

Supplementary Figure 1.

Supplementary Figure 2.

Supplementary Figure 3.

Supplementary Figure 4.

Supplementary Tables.

## Data Availability

The datasets used and/or analysed during the current study available from the corresponding author on reasonable request.
