# Author Correction: Geriatric nutritional risk index as a prognostic marker for patients with upper tract urothelial carcinoma receiving radical nephroureterectomy

**DOI:** 10.1038/s41598-024-81780-6

**Published:** 2024-12-06

**Authors:** Li-Wen Chang, Sheng-Chun Hung, Chuan-Shu Chen, Jian-Ri Li, Kun-Yuan Chiu, Shian-Shiang Wang, Cheng-Kuang Yang, Kevin Lu, Cheng-Che Chen, Shu-Chi Wang, Chia-Yen Lin, Chen-Li Cheng, Yen-Chuan Ou, Shun-Fa Yang

**Affiliations:** 1 Institute of Medicine, Chung Shan Medical University, No. 110, Sec.1, Jianguo N. Rd., Taichung, 40201 Taiwan, ROC; 2 Division of Urology, Department of Surgery, Taichung Veterans General Hospital, No. 1650, Sec. 4, Taiwan Boulevard, Taichung, Taiwan, ROC; 3 Department of Post-Baccalaureate Medicine, College of Medicine, National Chung Hsing University, Taichung, Taiwan, ROC; 4 Department of Medicine and Nursing, Hungkuang University, Taichung, Taiwan, ROC; 5 Department of Applied Chemistry, National Chi Nan University, Nantou, Taiwan; 6 School of Medicine, National Yang Ming University, Taipei, Taiwan; 7 Department of Medical Research, Taichung Veterans General Hospital, Taichung, Taiwan, ROC; 8 Department of Urology, Tungs’ Taichung MetroHarbor Hospital, Taichung, Taiwan, ROC; 9 Department of Medical Research, Chung Shan Medical University Hospital, Taichung, Taiwan

Correction to: *Scientific Reports* 10.1038/s41598-023-31814-2, published online 20 March 2023.

In the original version of this Article, an Affiliation for Shun-Fa Yang was omitted. The correct Affiliations are listed below.

Shun-Fa Yang.

- “Institute of Medicine, Chung Shan Medical University, No. 110, Sec.1, Jianguo N. Rd., Taichung, 40201, Taiwan, ROC.”
- “Department of Medical Research, Chung Shan Medical University Hospital, Taichung, Taiwan.”

The original Article has been corrected.

