# Supplementary material for: Geriatric nutritional risk index as a prognostic marker for patients with upper tract urothelial carcinoma receiving radical nephroureterectomy
Source: Sci Rep. 2023 Mar 20;13:4554. doi: 10.1038/s41598-023-31814-2 (PMC10027676; doi:10.1038/s41598-023-31814-2)
Supplement: Supplementary file 1 — Supplementary Legends. [file 41598_2023_31814_MOESM1_ESM.docx]

Supplementary Figure Legend

Supplementary Figure 1: Box plot showing the difference of GNRI index between patients with different pathological T stage (*p*=0.001).

Supplementary Figure 2: Box plot showing the difference of GNRI index between patients with different pathological N stage (*p*=0.001).

Supplementary Figure 3: Box plot showing the difference of GNRI index between patients with lymphovascular invasion or not (*p*=0.001).

Supplementary Figure 4: Box plot showing the difference of GNRI index between patients with surgical margin positive involvement or not (*p*=0.008).
