# Supplementary figures and images for: Geriatric nutritional risk index as a prognostic marker for patients with upper tract urothelial carcinoma receiving radical nephroureterectomy
Source: Sci Rep. 2023 Mar 20;13:4554. doi: 10.1038/s41598-023-31814-2 (PMC10027676; doi:10.1038/s41598-023-31814-2)

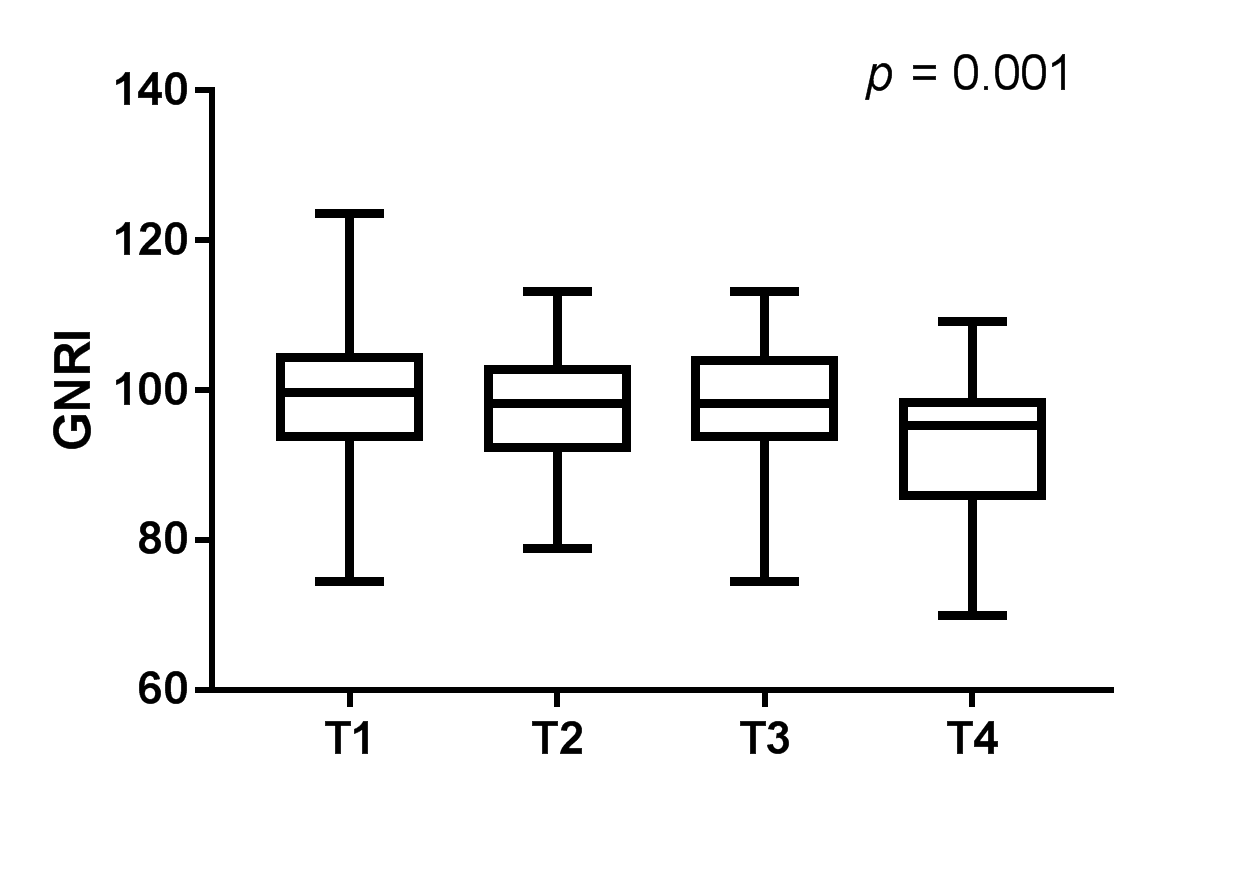

Supplement: Supplementary file 2 — Supplementary Figure 1. [file 41598_2023_31814_MOESM2_ESM.tif]

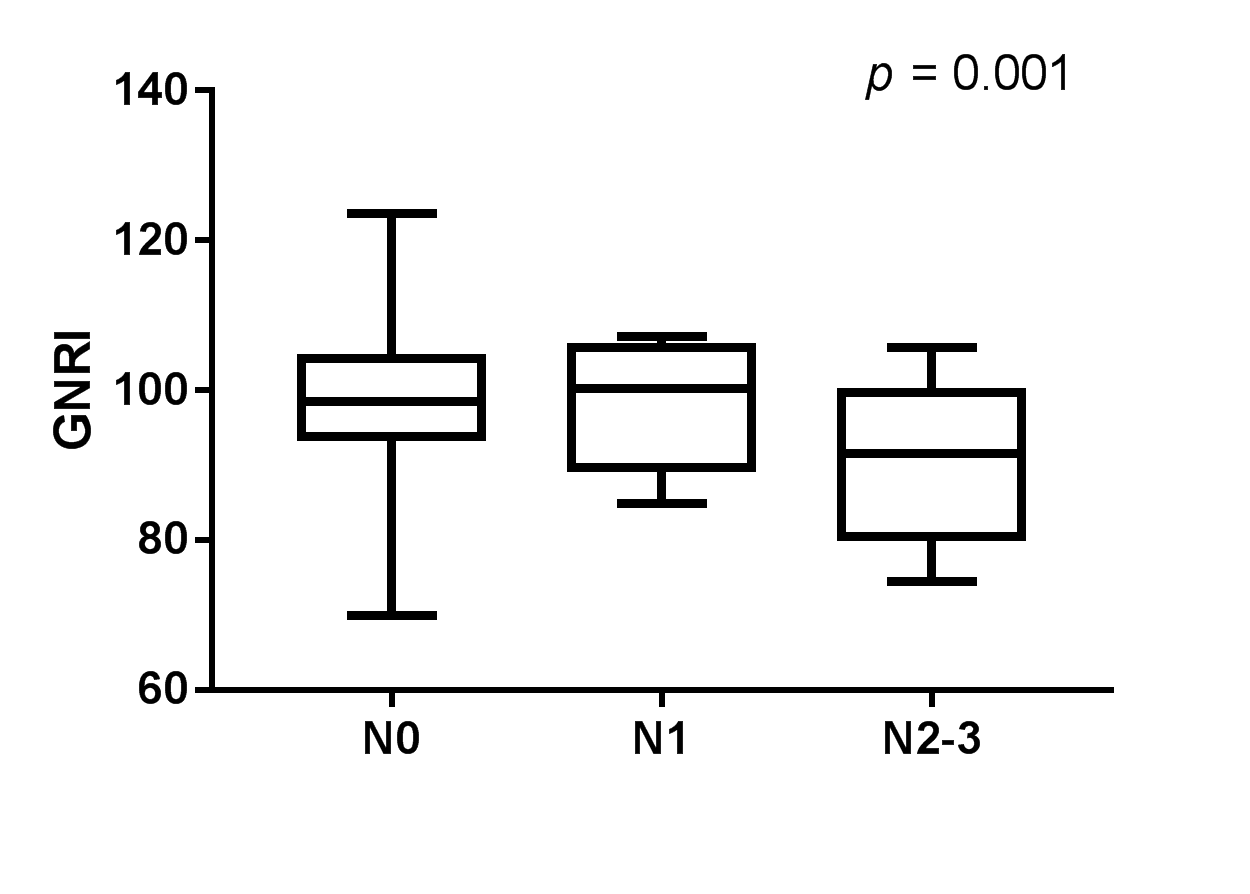

Supplement: Supplementary file 3 — Supplementary Figure 2. [file 41598_2023_31814_MOESM3_ESM.tif]

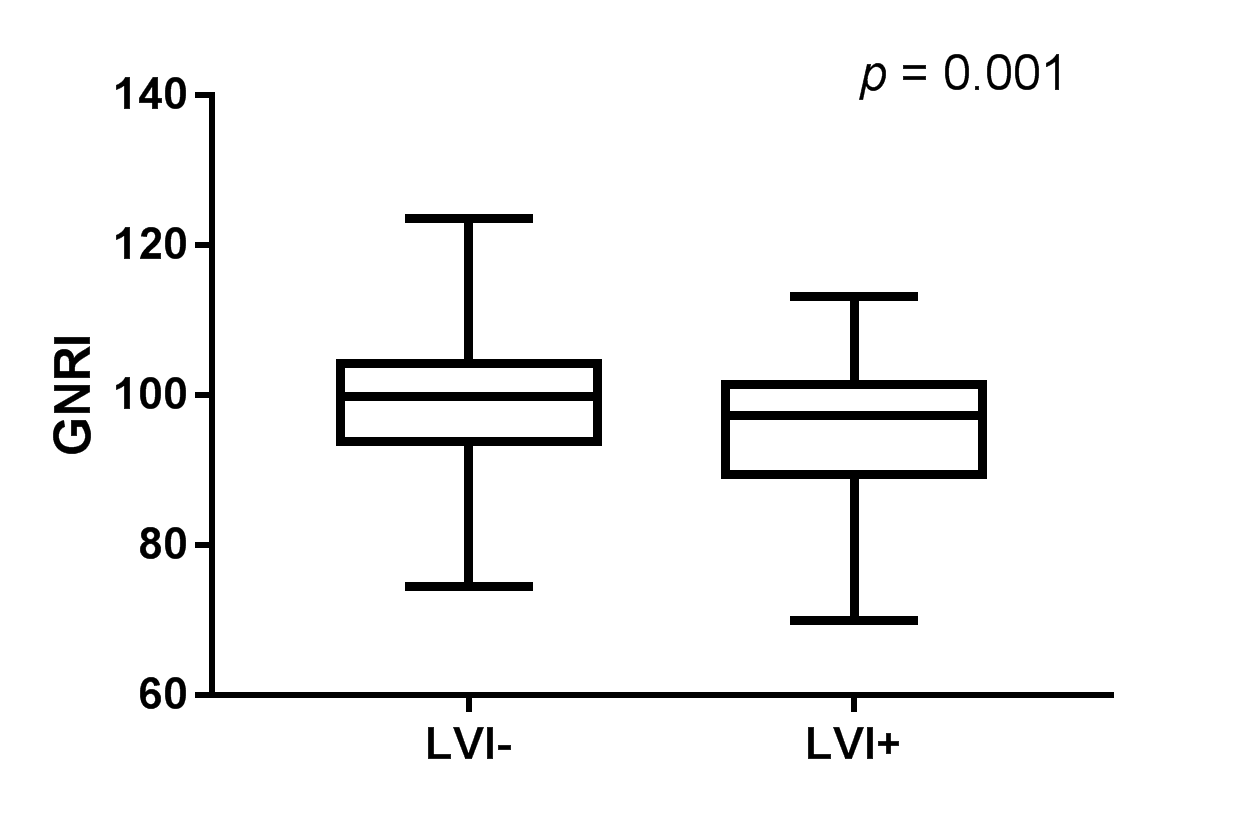

Supplement: Supplementary file 4 — Supplementary Figure 3. [file 41598_2023_31814_MOESM4_ESM.tif]

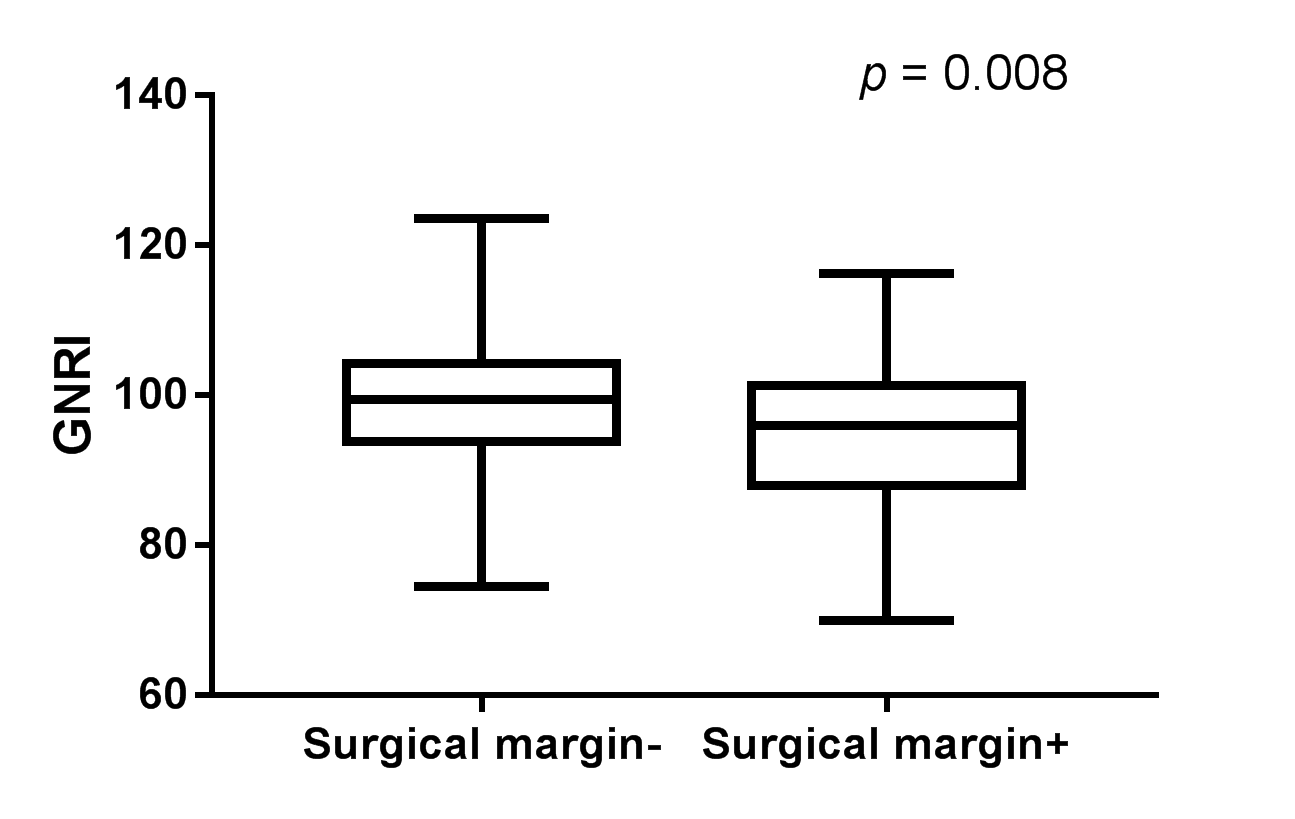

Supplement: Supplementary file 5 — Supplementary Figure 4. [file 41598_2023_31814_MOESM5_ESM.tif]
