# Supplementary material for: Geriatric nutritional risk index as a prognostic marker for patients with upper tract urothelial carcinoma receiving radical nephroureterectomy
Source: Sci Rep. 2023 Mar 20;13:4554. doi: 10.1038/s41598-023-31814-2 (PMC10027676; doi:10.1038/s41598-023-31814-2)
Supplement: Supplementary file 6 — Supplementary Tables. [file 41598_2023_31814_MOESM6_ESM.docx]

| Supplementary table 1. Demographic (N=488) | | | | | |
| --- | --- | --- | --- | --- | --- |
|  | GNRI ≤ 93.58 (n=120) | | GNRI > 93.58 (n=368) | | *P*-value |
| **Gender** |  |  |  |  | 0.776 |
| Male | 52 | (43.3%) | 152 | (41.3%) |  |
| Female | 68 | (56.7%) | 216 | (58.7%) |  |
| **Age** | 70.0 | (63.0-76.0) | 67.0 | (58.0-75.8) | 0.039* |
| **BMI (kg/m^2^)** | 22.9 | (19.8-25.0) | 24.2 | (21.9-26.2) | <0.001** |
| **Albumin (g/dL)** | 3.2 | (2.9-3.4) | 4.1 | (3.8-4.3) | <0.001** |
| **GNRI** | 87.9 | (84.9-90.8) | 101.3 | (97.9-105.7) | <0.001** |
| **Performance Status ECOG** |  |  |  |  | 0.003** |
| 0 | 15 | (12.5%) | 48 | (13.0%) |  |
| 1 | 71 | (59.2%) | 266 | (72.3%) |  |
| 2-4 | 34 | (28.3%) | 54 | (14.7%) |  |
| **Comorbidity** |  |  |  |  |  |
| CAD/HTN | 73 | (60.8%) | 225 | (61.1%) | 1.000 |
| DM | 30 | (25.0%) | 75 | (20.4%) | 0.346 |
| COPD/Asthema | 7 | (5.8%) | 10 | (2.7%) | 0.147 |
| CVA | 6 | (5.0%) | 13 | (3.5%) | 0.429 |
| Creatinine>1.5mg/dL | 36 | (30.0%) | 93 | (25.3%) | 0.368 |
| HBV or HCV carrier | 16 | (13.3%) | 40 | (10.9%) | 0.568 |
| Previous UCUB | 25 | (20.8%) | 62 | (16.8%) | 0.394 |
| Hydronephrosis | 12 | (10.0%) | 38 | (10.3%) | 1.000 |
| **Smoking status** |  |  |  |  | 0.593 |
| Never | 91 | (75.8%) | 270 | (73.4%) |  |
| Current/Former | 29 | (24.2%) | 98 | (26.6%) |  |
| **Preoperative renal function** |  |  |  |  | 0.225 |
| eGFR≥30 ml/min/1.73m^2^ | 85 | (70.8%) | 281 | (76.4%) |  |
| eGFR<30 ml/min/1.73m^2^ | 35 | (29.2%) | 87 | (23.6%) |  |
| **History of Uremia** |  |  |  |  | 1.000 |
| Negative | 102 | (85.0%) | 314 | (85.3%) |  |
| Positive | 18 | (15.0%) | 54 | (14.7%) |  |
| **Surgical modality** |  |  |  |  | 0.116 |
| Open | 21 | (17.5%) | 46 | (12.5%) |  |
| Transperitoneal laparoscopy | 92 | (76.7%) | 311 | (84.5%) |  |
| Retroperitoneoscopy | 7 | (5.8%) | 11 | (3.0%) |  |
| **Tumor location** |  |  |  |  |  |
| calyx | 28 | (23.3%) | 86 | (23.4%) | 1.000 |
| renal pelvis | 67 | (55.8%) | 230 | (62.5%) | 0.233 |
| promixal ureter | 47 | (39.2%) | 113 | (30.7%) | 0.109 |
| middle ureter | 31 | (25.8%) | 78 | (21.2%) | 0.351 |
| distal ureter | 26 | (21.7%) | 93 | (25.3%) | 0.499 |
| **Surgical margin** |  |  |  |  | 0.013* |
| Negative | 100 | (83.3%) | 338 | (91.8%) |  |
| Positive | 20 | (16.7%) | 30 | (8.2%) |  |
| **Pathological T** |  |  |  |  | 0.233 |
| T1 | 56 | (46.7%) | 194 | (52.7%) |  |
| T2 | 16 | (13.3%) | 43 | (11.7%) |  |
| T3 | 35 | (29.2%) | 110 | (29.9%) |  |
| T4 | 13 | (10.8%) | 21 | (5.7%) |  |
| **Pathological N** |  |  |  |  | 0.002** |
| N0 | 101 | (84.2%) | 344 | (93.5%) |  |
| N1 | 5 | (4.2%) | 11 | (3.0%) |  |
| N2-3 | 14 | (11.7%) | 13 | (3.5%) |  |
| **Tumor grade** |  |  |  |  | 0.140 |
| Low | 7 | (5.8%) | 38 | (10.3%) |  |
| High | 113 | (94.2%) | 330 | (89.7%) |  |
| **Concomitant CIS** |  |  |  |  | 0.170 |
| Negative | 95 | (79.2%) | 313 | (85.1%) |  |
| Positive | 25 | (20.8%) | 55 | (14.9%) |  |
| **Lymphovascular invasion** |  |  |  |  | <0.001** |
| Negative | 79 | (65.8%) | 302 | (82.1%) |  |
| Positive | 41 | (34.2%) | 66 | (17.9%) |  |
| **Adjuvant Chemotherapy** | 30 | (25.0%) | 87 | (23.6%) | 0.857 |
| **F/u Time (month)** | 24.0 | (11.5-40.8) | 41.9 | (27.7-65.0) | <0.001** |
| Chi-square test. Mann-Whitney Test, Median (IQR). **P*<0.05, ***P*<0.01 | | | | | |
| GNRI, Geriatric Nutritional Risk Index; BMI, body mass index; ECOG, Eastern Cooperative Oncology Group; HTN, hypertension; DM, diabetes mellitus; COPD, chronic obstructive pulmonary disease; CAD, coronary artery disease; HBV, Hepatitis B virus; HCV, Hepatitis C virus; UCUB, urothelial carcinoma in urinary bladder; eGFR, estimated Glomerular filtration rate; CIS, carcinoma in situ. | | | | | |

| Supplementary Table 2. Perioperative complications | | | | | |
| --- | --- | --- | --- | --- | --- |
|  | GNRI < 92 (n=102) | | GNRI ≥ 92 (n=386) | | *p-*value |
| Vascular injury | 3 | 2.9% | 7 | 1.8% | 0.506 |
| Wound infection | 3 | 2.9% | 8 | 2.1% | 0.162 |
| Ileus | 8 | 7.8% | 18 | 4.7% | 1.000 |
| All | 11 | 13.7% | 33 | 8.5% | 0.868 |
| Chi-square test. **P*<0.05 | | | | | |
